# The Clinical and Theranostic Values of Activated Leukocyte Cell Adhesion Molecule (ALCAM)/CD166 in Human Solid Cancers

**DOI:** 10.3390/cancers13205187

**Published:** 2021-10-15

**Authors:** Yiming Yang, Andrew J. Sanders, Q. Ping Dou, David G. Jiang, Amber Xinyu Li, Wen G. Jiang

**Affiliations:** 1 School of Medicine, Cardiff University, Henry Wellcome Building, Cardiff CF14 4XN, UK; yangy147@cardiff.ac.uk (Y.Y.); doup@karmanos.org (Q.P.D.); D.jiang1@nhs.net (D.G.J.); ds19875@bristol.ac.uk (A.X.L.); 2 Departments of Oncology, Pharmacology and Pathology School of Medicine, Barbara Ann Karmanos Cancer Institute, Wayne State University, Detroit, MI 48201-2013, USA; 3 Stoke Mandeville Hospital, Buckinghamshire Healthcare NHS Trust, Aylesbury HP21 8AL, UK

**Keywords:** activated leukocyte cell adhesion molecule, ALCAM, CD166, cancer, prognosis, survival, therapeutic targets

## Abstract

**Simple Summary:**

ALCAM (activated leukocyte cell adhesion molecule) is an important regulator in human cancers, particularly solid tumours. Its expression in cancer tissues has prognostic values depending on cancer types and is also linked to distant metastases. A truncated form, soluble form of ALCAM (sALCAM) in circulation has been suggested to be a prognostic indicator and a potential therapeutic tool. This article summarises recent findings and progress in ALCAM and its involvement in cancer, with a primary focus on its clinical connections and therapeutic values.

**Abstract:**

Activated leukocyte cell adhesion molecule (ALCAM), also known as CD166, is a cell adhesion protein that is found in multiple cell types. ALCAM has multiple and diverse roles in various physiological and pathological conditions, including inflammation and cancer. There has been compelling evidence of ALCAM’s prognostic value in solid cancers, indicating that it is a potential therapeutic target. The present article overviews the recent findings and progress in ALCAM and its involvement in cancer, with a primary focus on its clinical connections in cancer and therapeutic values.

## 1. Introduction

ALCAM (activated leukocyte cell adhesion molecule), also known as CD166, was discovered more than two decades ago and has been well established as a pivotal cell adhesion protein mediating both homotypic and heterotypic cell-cell adhesions in the body. Although it was initially thought to be key to leukocyte and leukocyte-endothelial interactions, ALCAM has now been found to be present ubiquitously in the body and almost all cell types. The physiological and pathological implications of ALCAM have also been recognised well beyond classical cell adhesion. One area that has been increasingly explored is its impact on cancer. The present article aims to summarise the key findings of ALCAM in the area of cancer and will focus on the clinical, prognostic, and therapeutic aspects of ALCAM in cancer.

## 2. ALCAM, Its Discovery and Cellular Connections

**Figure 1:**
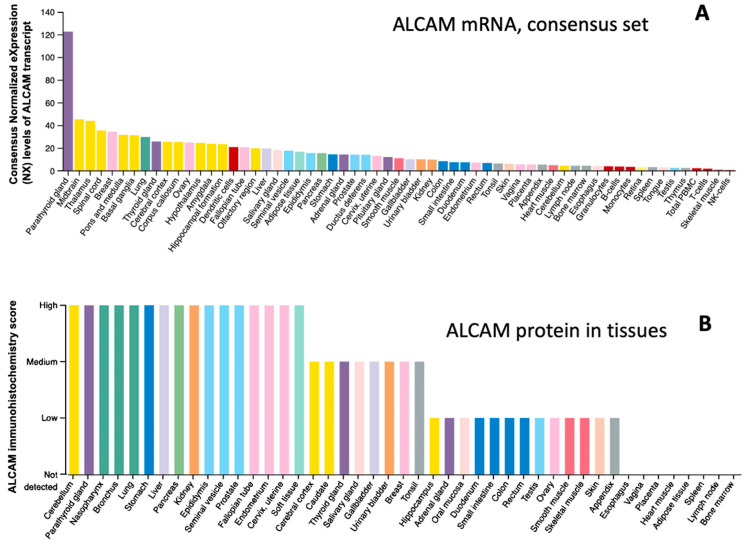
Levels of expression of ALCAM mRNA and protein in human tissues [3] (www.proteinatlas.org) (accessed on 22 September 2021). (**A**): Expression of ALCAM mRNA levels (consensus normalised expression (NX)) in tissues of consensus cohort for 55 tissue types and 6 blood cell types with a combination of data from the three transcriptomics data sets (HPA, GTEx, and FANTOM5). The mRNA profile indicated that nerve tissues, mammary tissues, lung, thyroid, and parathyroid glands, ovary tissue tend to have high levels of ALCAM mRNA expression, whereas haematopoietic cells, lymphoid tissues, skins or tissues with squamous cell types, and digestive system generally expressed low levels of ALCAM mRNA. (**B**): ALCAM protein expression levels in tissues (immunohistochemical staining) from the HPA data set. At the protein level, brain tissues, respiratory system, parathyroid, pancreas, male reproductive tissues, prostate, and kidney have high levels. Lymphoid tissues, muscles, skin, tissues with squamous cell cells (vagina, oesophagus) have low to negligible levels of ALCAM protein.

ALCAM was discovered in 1995 as a ligand for CD6 [1,2]. By transfecting CHO cells to overexpress CD6, it was found that a protein, subsequently named ALCAM, supported the thymocyte-thymic epithelial interactions [1]. ALCAM was subsequently purified and identified as a type I transmembrane protein of 65kDa or 100–105 kDa as the result of glycosylation. The *ALCAM* gene is located to 3q13.1-q13. As an interacting partner for CD6 and being highly expressed in activated leukocytes, ALCAM has since been found to be expressed ubiquitously in the majority of cell and tissue types [3] (Figure 1). Overall, central nervous tissues, thyroid, and parathyroid tissues, pancreas, tissues of the respiratory system, and prostate tend to have high levels of ALCAM at both mRNA and protein levels. Instead, haematopietic cells, lymphoid tissues, tissues with squamous cells, including skin, vagina, and muscle tissues, have lower levels of ALCAM (Figure 1A,B).

## 3. ALCAM Expression Pattern in Cells and Tissues and Influence on Other Genetic and Biochemical Events

**Figure 2:**
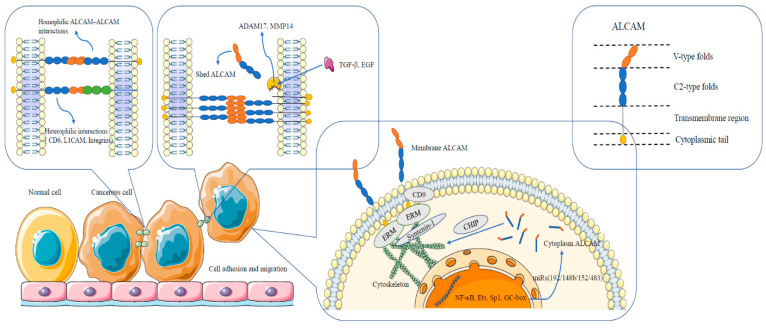
Diagrammatic representation of ALCAM structure and interactions between and within cells. ALCAM, a transmembrane protein, consists of both C2 and V type fold extracellular domains important in its clustering and interactions with neighbouring cells, through either ALCAM-ALCAM or ALCAM-CD6, etc. interactions. Cleavage of ALCAM from the membrane can occur by the proteolytic action of proteases such as ADAM17 or MMP14, releasing or shedding the indicated domains from the cell’s surface. ALCAM intracellular interactions have also been indicated.

It has been well established that ALCAM interacts in a homotypical and heterotypical manner when mediating cell adhesion both between the same cell types and in different cell types. It is also established that ALCAM, via its intracellular domain, interacts with the ERM family of proteins (Ezrin, moesin, and radixin), which confers cell adhesion mechanism (Figure 2). An important finding over the past decade is that ALCAM could be shed from the cell surface by enzymatic cleavage, producing a new form of the protein, soluble ALCAM (sALCAM). The cleavage of the ALCAM protein occurs mainly at its ectodomain region, and some well know ALCAM-cleaving enzymes include ADAM17 and MMP14, as illustrated in Figure 2. The key biochemical and cellular impacts of ALCAM have been recently summarised by von Lersner et al. [4]; the authors have documented the genes and proteins likely to be influenced by ALCAM. The ALCAM-mediated signal pathways in different cells are illustrated in Figure 2.

## 4. ALCAM Protein as a Receptor for Tumour Cell Homing

ALCAM has been well established as a cell adhesion protein from the time of its discovery. However, the recognition that ALCAM may serve as a pivotal receptor for a cancer cell to seek its metastatic destination places it as an important player in the ‘seed and soil’ theory of cancer metastasis proposed more than a century ago [5] in which cancer cells referred as ‘seed’ of metastasis have their ability to identify the suitable tissues/organs (soil) to establish distant metastases. The theory has stood for test over the century and has inspired new insights, including the identification of ‘homing receptors’ for the process [6,7]. Ruma et al. [8] have shown that ALCAM acts as one of the ‘soil’ sensor receptors for S100A8/A9/S100P, which in turn serve as ‘soil signals’. Other ‘soil sensor receptors’ include MCAM (melanoma cell adhesion molecule), NPTN-beta (neuroplastin beta), EMMPRIN (extracellular matrix metalloproteinase inducer), and TLR4 (toll-like receptor 4) [8]. Another study has demonstrated that cell surface ALCAM is endocytosed for recycling by its expressing cell that requires endophilin, particularly the A3 isoform [9]. The endocytosis process of ALCAM appears to be driven by galactin-8 of the extracellular origin. The authors of this study have shown that blocking endophilin-A3 markedly increased the amount of cell surface ALCAM and ALCAM-mediated cell adhesiveness [9].

## 5. ALCAM Expression and Its Functional Influence in Cancer Cells

The role of ALCAM in cancer cells has been an interesting topic of debate. For example, in Benzo[a]pyrene transformed human bronchial epithelial 16HBE cells, knockdown of ALCAM rendered the cells with lower growth and colony formation potentials, but in contrast, with an increase in cellular migration and lung metastasis in vivo [10]. Again in lung cancer cells, knocking down of ALCAM resulted in a reduction in adhesion to brain endothelial cells both in culture and shear stress conditions [11]. Interestingly, Ezrin and syntenin-1 have been shown to be linker proteins for ALCAM to anchor to the cytoskeleton [12]. Inhibition of the degree of glycosylation of ALCAM by kifunensine results in the shift of ALCAM from the membrane fraction to the cytoplasmic fraction, which also coincides with the increase in cell migration of ovarian cancer cells [13]. The change of cellular fraction of ALCAM in cancer is intriguing, and its biological and clinical impact is yet to be fully understood. Kahlert et al. [14] have reported that patients with pancreatic cancer, who primarily show cytoplasmic/non-membranous ALCAM staining, tend to have better survival than those with membranous staining. ALCAM expression levels (mRNA and protein) appear to be induced by the presence of Wnt5a in breast cancer cells [15], an expression event that is linked to the migration of breast cancer cells. Similarly, activation of SHH (sonic hedgehog) has been shown to increase the protein half-life of ALCAM in oral SCC cells [16], arguing a protein-level regulation of ALCAM in cancer. Work by Fernandez et al. and Ferragut et al. [17,18] has shown that ALCAM, via its glycosylation, works in orchestration with Galectin-8, a portraited internal ligand for ALCAM, in triple-negative breast cancer cells [18]. Knockdown of ALCAM and/or galectin-8 was found to reduce levels of the matrix adhesiveness, proliferation, cell-cell adhesion of breast cancer cells, and tumour growth in vivo [18]. This was probably attributable to the sialylation of ALCAM on the cell surface. ALCAM expression can also be regulated by microRNAs. For example, microRNA-192, miR-148b-3p, miR-152, and miR-483-5p have been shown to be able to suppress the expression of ALCAM in tumour cells, including gastric, pituitary, and liver cancer cells, possibly by targeting the 3′-UTR binding site of the transcription region of ALCAM gene [19,20,21,22].

ALCAM expressed on mesenchymal stem cells has also been demonstrated to assist the cells to migrate toward tumours, such as glioma in the brain, a tumour tropic movement [23]. Furthermore, ALCAM, in line with E-cadherin but in contrast to beta-catenin and CD44, appears to be an EMT epithelial phenotypic molecule in response to TWIST induced EMT (epithelial-mesenchymal transition) in colorectal cancer cells, and the response seems to be dependent on the microsatellite instability of the cells [24]. In uveal melanoma cells, knockdown of ALCAM from the cells resulted in reduced cellular migration and invasiveness and disrupted cell-cell junctions [25]. At the junction area, ALCAM co-localises with beta-catenin and N-cadherin (CDH2), indicating a key role in forming adherens junctions, a mesenchymal phenotype of the cells.

Thus, the expression and the pattern of expression of ALCAM in cancer have an important bearing on the function of cancer cells. It has been proposed that both CD166 and its interactive partner proteins may be suitable for therapeutic targeting in a direct manner (targeting CD166) or indirect manner (targeting the CD166 interacting partners). For example, in lung stem-like cancer cells that display positivity of CD166, together with CD44 and EpCAM, the cells were found to be more sensitive to NFκB inhibitors for suppressing the expression of CD166, suggesting a strategy to target this small population of cancer cells for treatment [26,27]. The importance of CD166 in leukaemia has been well demonstrated by a study in which blocking CD166-ILT3 interactions with an ILT3-Fc dramatically reduced proliferation and growth of leukaemia cells and increased the survival of tumour-bearing mice (in both H9 and Jakart models) [28]. CD6 is a well-established binding partner for CD166 [1,2] and has been proposed to be a useful therapeutic target in conditions such as autoimmune diseases [29]. It was recently established that CD318 is also a binding partner for CD6 [30]. This is very interesting since CD166 is widely expressed in haematopoietic cells and also in almost all other cell types, whereas CD318 is only expressed in non-haematopoietic cells, parting way for cell-specific targeting. Furthermore, L1CAM (L1 cell adhesion molecule) on breast cancer cells has been shown to interact with ALCAM on endothelial cells promoting tumour-endothelial interactions [31]. Additionally, 14-3-3ζ and 14-3-3σ have also been identified as interacting partner proteins for ALCAM in oral SCC (squamous cell carcinoma) cells [32].

## 6. ALCAM Expression in Human Solid Malignant Tumours and the Clinical/Prognostic Significance

**Figure 3:**
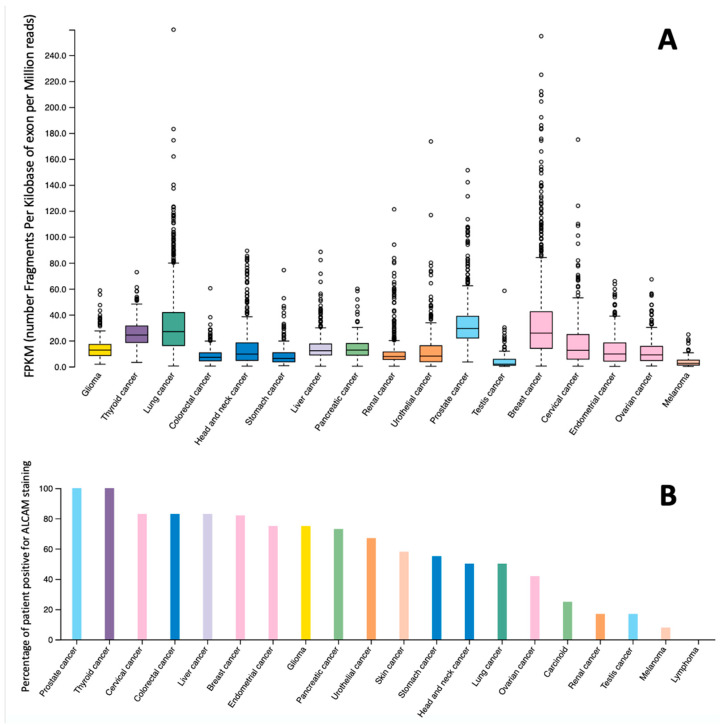
Expression pattern of ALCAM mRNA (**A**) and ALCAM protein (**B**) in various human cancers [33] (www.proteinatlas.org) (accessed on 22 September 2021).

As with its expression pattern in normal tissues (seen in Figure 1), ALCAM also displays a diverse expression pattern in solid cancers. Figure 3 demonstrates the available information of the overall expression pattern of ALCAM in various cancer types at mRNA and protein levels, in which a number of endocrine-related cancers, including breast, prostate, thyroid, and lung cancers, have high expression levels [33]. This is interesting as these 4 cancer types have a high tendency for bone metastasis. It has been reported previously that ALCAM was indeed a protein linked to bone metastasis [34,35]. The following sections have summarised the clinical and pathological impacts of ALCAM in cancer types that are highlighted in Table 1.

### 6.1. Pancreatic Cancer

In a small size study of patients with pancreatic ductal adenocarcinoma, Amantini and colleagues [61] have shown that when circulating cancer cells had high levels of ALCAM, patients tend to have significantly shorter survival. Another early study involving 97 patients with pancreatic cancer showed that patients who died of pancreatic cancer tend to have stronger ALCAM staining [14]. Together with chemotherapy and tumour staging, ALCAM appears to be an independent survival indicator of the patients. Although it has been shown that, in a relatively small cohort of PNET (pancreatic neuroendocrine tumours), raised tissue levels of ALCAM are positive factors for both recurrence-free survival and disease-specific survival [97,98], the same authors, in a larger pancreatic cancer cohort (*n* = 264) did not find a connection between tissue ALCAM and the survivals [62]. Furthermore, in a small cohort of pancreatic cancer, Zhang et al. [99] have shown that pancreatic cancer cells in the tumour tissues stained rather weakly for ALCAM. However, the authors showed that it was the stella cells inside and around tumours are highly positive for ALCAM staining [99]. Similarly, it has been shown that only 12% of pancreatic tumours stained positive for ALCAM [63]. Fujiwara went ahead to show that pancreatic cancer cells with negative/low ALCAM tend to be highly invasive and more tumorigenic in vivo [63]. Overall, ALCAM appears to be an indicator for poor outcome of the patients with pancreatic with the exception of pancreatic neuroendocrine tumours, which otherwise show an opposite link, namely high levels of ALCAM indicates a favourable outcome. The latter observation is interesting since the neuroendocrine tumours types appear to echo other endocrine tumours, namely breast, prostate, and thyroid cancers (refer to Section 6.4).

### 6.2. Colorectal Cancer

There have been some extensive studies on the role of ALCAM in colorectal cancer. This was reflected in a subsequent meta-analysis [100]. This meta-analysis revealed that high levels of ALCAM in colorectal cancer are associated with poor overall survival, nodal status, tumour grade, and distant metastasis [100]. Shedding of ALCAM, as assessed by levels of intracellular ALCAM and extracellular domain of ALCAM in immunocytochemistry, was markedly higher in patients with colorectal cancer and is progressively increased with tumour staging. This rise is associated with both overall survival and disease-free survival [40]. However, tumour tissues were found to have high levels of ALCAM protein and transcript, and the high levels were associated with shorter survival of the patients [40]. Badic et al. have also shown that the ALCAM gene transcript was highly raised in colorectal cancer tissues compared with normal control tissue [42].

Park et al., using a matched cohort of colon cancer with microsatellite instability (MSI) and microsatellite stable colorectal tumours, found no difference in ALCAM expression between the groups [101]. Work by Ribeiro et al. showed that in colon cancer tissues, ALCAM-negative staining is significantly correlated with lymph node metastasis, an increased risk of 2.7 times that of ALCAM-positive tumours. However, this connection of negative ALCAM and nodal involvement is greatly enhanced, by a factor of 8, when the tumours also had KRAS mutation [38]. Another study, using a cohort of patients with colon cancer (*n* = 234) who received 5-FU (fluorouracil)-based chemotherapies after surgery, indicated that ALCAM polymorphisms (G > A) alone or with polymorphisms of LGR5 (T > C) and CD44 (C > T) and ALDH1A1 (G > C) are important factors in predicting extended time to recurrence and, in particular a combination of LGR5, CD44 and ALDH1A1 (although only LGR5 polymorphisms is a factor in predicting resistance to 5-FU-based therapies), not ALCAM [102]. In a rectal cancer study containing patients who received 5-FU-based neoadjuvant therapies, it was indicated that those with high pre-therapy ALCAM tumours had a significantly long disease-free survival, independent of other factors [39]. Thus, it is plausible that ALCAM is an indicator of poor clinical outcomes of patients with colorectal cancer. The finding that the presence of RAS mutation together with ALCAM expression contribute to the involvement of lymph nodes further suggested that ALCAM may be a suitable prognostic indicator and, in the selective group, may be a therapeutic indicator to chemotherapies and targeted therapies to RAS.

### 6.3. Gastric Cancer

In a cohort of 142 patients with gastric cancer, the positive membrane pattern of ALCAM was found to be linked to a significantly shorter survival of the patients, and both membrane and cytoplasmic staining of ALCAM was linked to vascular invasion and nodal metastasis [91]. In a comprehensive study with gastric cancer patient cohorts, ALCAM protein staining and transcript expression in tumour tissues were found to be highly raised. Both the membranous staining and messager level of ALCAM was seen in tumours with lymphatic invasion. Although membranous staining of ALCAM is linked to tumour staging and shorter overall survival, the cytoplasmic presence of ALCAM protein seems less prominent in the relationship with clinical outcome and survival of the patients [92]. The study has shown that circulating ALCAM in patients is at significantly high levels compared with control individuals and those with pre-cancerous conditions. The latter two themselves also showed detectable levels of ALCAM in their circulation, together indicating that circulating ALCAM is an important indicator of gastric cancer but lacks a clear diagnostic value. Overall, studies on ALCAM in stomach malignancies are less intensive, and cohorts studies were relatively small. More comprehensive investigations of this nature are required in the future.

### 6.4. Breast Cancer

A large amount of research has focused on ALCAM in breast cancer development and progression. In early studies by Hein et al. [103], it was shown that ALCAM staining in primary breast tumours is associated with nodal involvement and the presence of cancer cells in bone marrow, and indeed with both shorter overall and recurrence-free survival of the patients. In a comprehensive and classical immunohistochemical analysis of 2197 breast tumours (TMA), Burandt and colleagues analysed the ALCAM staining pattern and found that most tumours stained positive and strong for ALCAM [60]. Almost all the histological types had strong ALCAM staining, except the medullary subtype, with over 40% showing negative staining. High-grade, ER-positive, and PR-positive tumours tend to be more intensive in staining, and that loss of ALCAM staining was associated with significantly shorter overall and disease-free survival of the patients. Similarly, the loss/reduction in membranous ALCAM is observed by Tan et al. [104]. In their study, the authors compared the pattern of staining of ALCAM in two different ethnic populations of the U.S. patients, namely African American (*n* = 78) and Caucasian American (*n* = 95). Although relatively small in the study cohorts, it does reveal a rather different staining pattern between the two groups, and specifically, tumours from African American patients is four-times likely to have reduced/loss of membranous ALCAM staining than that from the Caucasian patients, yet, the cytoplasmic staining of ALCAM does not differ between the two groups. The relationship between ALCAM with other clinical and pathological factors are otherwise similarly impacting both ethnic groups [104]. Furthermore, a study by Ihnen et al. [55], containing 481 breast tumours has discovered that a subset of the patients with ALCAM(low)/Osteopontin(High)/ER(negative)/Her2(negative) tumours had markedly shorter disease-free and overall survival and that the combination is a strong independent prognostic indicator. Additionally, other work has demonstrated that patients that had high ALCAM and high MAN1A1 had significantly longer disease-free survival than those with low ALCAM and high MAN1A1 [57]. Furthermore, Jeong et al. demonstrated that breast tumour tissues with high levels of ALCAM gene methylation had been observed to have low levels of ALCAM transcripts [58], suggesting a possible inhibitory regulation. Similarly, the authors show that ALCAM transcript levels in tumours are positively correlated with a number of inflammatory markers, including TNFα (tumour necrosis factor alpha), IL4 (interleukin 4), and NF-κB, and that Her2-positive and high-grade tumours had high ALCAM proteins and Luminal-A tumours had the strongest ALCAM staining [58]. ALCAM has also been indicted for having a prognostic value in combination with other molecules. For example, from multiple databases, Dai et al. [105] have discovered a four-gene signature, including ALCAM, that had a significant prognostic value in patients with breast cancer.

Polymorphisms have been reported as a frequent feature of breast cancer. In a cohort of 1033 breast cancers (compared with 1116 control subjects) from a Chinese study, two single-nucleotide polymorphisms (SNPs) of the *ALCAM* were identified, rs6437585 (C/T) and rs11559013 (A/G) [106]. The presence of rs6437585 in the patients was associated with a significantly raised risk of developing breast cancer, a phenomenon not seen with rs11559013. In a Swedish cohort of 783 breast cancer, 2 of the *ALCAM* single-nucleotide polymorphisms (rs1044243 and rs115) were found to be associated with the survival of the patients [107]. Low and colleagues, in their genome-wide association studies (GWAS) [56], discovered that 3q13.11 (*ALCAM*), together 21q22.12 (*CLIC6-RUNX1*), two novel loci, are potential susceptibility indicators in Japanese breast cancer patients.

From the rather wide and comprehensive studies on breast cancer, it would appear that ALCAM is a favourable prognostic factor for the patients, in clear contrast to those with colorectal and gastric cancers. This connection in breast cancer is further supported by findings that low levels of ALCAM also appear to facilitate bone metastasis of the patients [24,25]. The reasons underlying the contrasting connection of ALCAM and clinical outcomes in breast cancer and gastrointestinal cancer are unclear. However, endocrines and hormones may be one of the contributing factors. In breast cancer, when hormone receptors ER and Her2 are low or negative, ALCAM appears to be more prominent in its value in predicting the prognosis. As it will be discussed in a later section of this article, other endocrine-related tumours, namely prostate cancer, thyroid cancer, to a degree ovarian cancer, together with the aforementioned pancreatic neuroendocrine tumours (PNET) [34,35], also bear a similar prognostic feature to breast cancer. This ALCAM-hormone/endocrine link will be a promising area to explore in the future.

### 6.5. Sarcomas

In a cohort of 98 Ewing sarcomas, ALCAM (mostly membranous) was found in the majority of the tumours, and patients with high levels of ALCAM had favourably MFS (metastasis-free survival) [93]. Similarly, it was also found that HDGF (hepatoma-derived growth factor), identified as an ALCAM transcription suppressor, when highly expressed together with lower ALCAM expression, was associated with worse patient outcomes.

### 6.6. Myeloma

In multiple myeloma (in vivo model), the loss of ALCAM was seen together with increased survival, reduced osteolytic lesions, and changed bone modelling, an event thought to be due to ALCAM-mediated osteoclastogenesis via an altered balance of RANKL (receptor activator of nuclear factor kappa-B ligand) and osteoprotegerin [108]. In myeloma cells, ALCAM is a downstream response protein to migration inhibitory factor (MIF) and an important factor in the interaction between myeloma cells and bone marrow stromal cells [109].

### 6.7. Prostate Cancer

A number of studies have explored the impact of ALCAM expression in prostate cancer. In a large cohort of tissue microarray (*n* = 2390), ALCAM was found largely membranous stained in prostate cancers (69.9%). Cytoplasmic staining was seen as a unique pattern in a small proportion of the samples. It is the membrane staining in prostate tumours that is associated with a more favourable clinical, pathological, and outcome status of the patients. Instead, cytoplasmic staining does not appear to suggest any significant meaning [76]. Prostate cancer cells in the tumour tissues displayed both membrane and cytoplasm staining. By using the TCGA database, Hansen et al. reported a significant link between high levels of ALCAM transcript and shorter survival of the patients, with the high levels particularly seen in metastatic prostate cancers [78]. Although the number of studies in prostate cancer is small, it does appear to point to the importance of the cellular location of ALCAM in prostate cancer cells in tumour tissues, arguing a role of ALCAM-mediated cell adhesion and the integrity of membrane ALCAM in the potential tumour suppressive function of the protein.

### 6.8. Thyroid Cancers

Miccichè et al. [110] has reported, in early findings, that thyroid cancer expressed ALCAM on the membrane and cytoplasmic region and is able to shed it from the cell membrane [110]. Total ALCAM in poorly differential thyroid tumours was found to be markedly lower than those with well/moderately differentiated tumours, and the reduction was associated with distant metastasis and shortened survival (6 years vs. 13.7 years, low vs. high cytoplasmic ALCAM, respectively) [74].

### 6.9. Ovarian Cancer

A recent study has shown that ALCAM expression has a connection with the survival of patients with ovarian cancer, and this connection is dependent on the status of a mannosidase MAN1A1 (Mannosidase Alpha Class 1A Member 1) [13]. While MAN1A1 itself has no significant connection with the survival, when the patients were grouped into those with high and low expression levels of MAN1A1, the levels of ALCAM in the subgroups appear to have a significant value in evaluating patient’s outcome. Those patients with high MAN1A1 and ALCAM-positive had significantly shorter relapse-free survival than those with high MAN1A1 and low or no ALCAM. In sharp contrast, those patients who had low MAN1A1 and high ALCAM had significantly longer survival than those with low MAN1A1 and low ALCAM. Together with in vitro observations that inhibit MAN1A1 by kifunensine, a glycosylation inhibitor of MAN1A1, resulted in a shift between the cell surface and cytoplasmic fractions of ALCAM in ovarian cancer cells [13], it collectively indicates that the degree of glycosylation of ALCAM plays a key role in its function and clinical manifestation. Additionally, the suppression of MAN1A1 by kifunensine in ovarian cancer and breast cancer cells [13,57] results in the cancer cells being more adhesive to endothelial vascular cells and more aggressive.

### 6.10. Endometrial Cancers

Of the endometroid endometrial cancers, over three-quarters of tumours stained positive for ALCAM, mostly membranous and cytoplasmic in location, and the positivity was linked to a shorter recurrence-free survival [68]. The study has shown that ALCAM positivity and shorter recurrence-free survival are particularly prevalent in the early stage of endometrial cancers by acting as an independent prognostic indicator [68]. The same authors subsequently reported that most of the ALCAM protein stained in endometrial cancers was on membranes (67.4% positive and 32.6% negative). The positivity of ALCAM staining was found to be an independent factor of tissue invasion of cancer [69]. The observation that the levels of soluble ALCAM in the uterine aspirate fluids of the patients correlated well with MMP9 staining in tumour tissues indicated the possibility of ALCAM shedding from tumours by the metalloproteinases [69].

### 6.11. Hepatocellular Carcinoma

In a cohort study of recurrent hepatocelluar carcinoma, the ALCAM-positive group was found to take a longer time to recurrence than those with ALCAM-negative tumours. This coincided with the finding that patients who had longer times to recurrence (>2 years) had lower levels of miR-483-5p than those that had shorter times to recurrence (<2 years), supporting the hypothesis that miR-483-5p is probably a suppressor of ALCAM [22].

### 6.12. Neurological Cancers

Neural tissues arguably have the highest level of ALCAM, as portrayed in Figure 1. The role of ALCAM in neurological malignancies has been well explored. Work undertaken by Allmendinger et al. [111] has measured ALCAM protein levels in a rather large group of neurological tumours (*n* = 339) and found that 93% of glioblastomas, 85% of lower-grade ependymomas, 83% of diffuse astrocytomas, and 68% of anaplastic ependymomas stained positive for ALCAM, although the staining tended to be weaker than normal control tissues (*n* = 105) [111]. In addition, patients with glioblastoma with high levels of ALCAM protein staining have shorter overall and disease-free survival [84]. In neuroblastoma, the cellular location of ALCAM seems important because ALCAM on the cellular membrane of neuroblast or missing from the neuropil area of the cells is an indicator for relapse of the patients [112]. In a cohort of 45 medulloblastoma tumours, ALCAM positivity was observed in only 18% of the tumours, with positive tumours mainly seen in the WNT group and the SHH group of the patients [85]. The study has found that ALCAM positivity was significantly linked to the status of CNNTB1 and the presence of nuclear beta-catenin in the medulloblastoma tumours.

In intracranial meningioma, ALCAM and PECAM1 (platelet and endothelial cell adhesion molecule 1), as well as selectins, stained highly positive compared to normal control tissues [82]. However, in this study, the two tissue types were different in nature, namely tumour tissues after surgery while normal control tissues from autopsies. In a cohort of 66 infantile neuroblastoma, weak ALCAM staining was linked to a shortened overall survival and recurrence-free survival in the infantile patients. It is interesting to note that ALCAM levels are linked to the status of Myc amplification of the tumours [83]. In another study to search for proteomic changes in chordoma, ALCAM was identified to be one of the lead upregulated proteins in recurrent chordoma compared with the primary chordoma tumours [113].

### 6.13. Lung Cancer

Berg et al. [114] have compared the levels of circulating ALCAM in three groups of patients, namely those with lung cancer, with chronic obstructive pulmonary diseases (COPD), and those with both conditions concurring. The study revealed that patients with COPD had significantly higher levels of circulating ALCAM compared to those with lung cancer and concurrent diseases, the latter two groups showing no difference. However, this study did not show any relationship between the circulating ALCAM and the outcome of the patients. It did not have a suitable control population to compare with either, which is essential to be included for future studies.

### 6.14. Mesothelioma

A total of 55% of 47 malignant mesotheliomas were positively stained for ALCAM [95]. In a cohort of 175 pleural mesotheliomas, 25% of the tumours showed positive staining of ALCAM, mostly membraneous [81]. However, patients with membranous ALCAM-positive mesotheliomas had a marked shorter survival compared with those with either negative ALCAM or cytoplasmic ALCAM, arguing membranous ALCAM as an independent prognostic indicator in this tumour type.

### 6.15. Oesophageal Cancer

ALCAM staining was detected in the majority of primary oesophageal adenocarcinoma and squamous cell carcinomas (71% of 299), metastatic lymph nodes (76% of 147), and metastatic tumours (80% of 46), and raised ALCAM levels were associated with recurrence-free and overall survival [44]. In human oesophageal squamous cell carcinoma (SCC), tumour tissues were found to have a high degree of staining of ALCAM, which was linked to tumour grade, TNM, and survival [45].

### 6.16. Head and Neck SCC (HNSCC) and Laryngeal Squamous Cell Carcinoma (SCC)

Tissues bearing squamous cell lineages, such as skin and vagina, generally have low levels of ALCAM (Figure 1). The same appears to be true for the squamous cells of the head, neck, and nasopharyngeal tissues. Thus, there has been a great interest in investigating ALCAM in these tumours. It has been observed in HNSCC that high levels of ALCAM staining, compared to those with lower staining, were associated with shorter survival. Additionally, recurrent HNSCC was found to have significantly higher ALCAM than the primary tumours [49]. In a tissue array of 400 HNSCC, Clauditz et al. have shown that 70% of HNSCC tumours stained positive for ALCAM, including 12.4% membranous, 40.1% cytoplasmic, and 17.9% mixed membranous/cytoplasmic. However, the study failed to show any link to clinical and pathological factors and the survival of the patients [48].

Using gene microarray analysis, Nicolau-Neto and colleagues [47] have shown that ALCAM is a significant and independent marker for the clinical prognosis of the patients with impressive specificity (94.7%) and sensitivity (93.1%). At the protein level, ALCAM, which was largely confined to the cell membrane, was detected in over 72% of the tumours, and high ALCAM protein levels were linked to a short survival of the patients [47].

Overall, it appears in squamous cell cancers, to the least in the head, neck, and pharynx tissues, high levels of ALCAM indicate a poor outcome of the patients.

### 6.17. Cutaneous Melanoma

A previous study found that invasive melanoma had strong ALCAM staining compared with non-invasive and nevi tissues [115]. Similarly, work by Djirackor et al. [116] has shown that, by using a cell line-based model and a TCGA cohort, high levels of ALCAM are associated with a poor clinical outcome. In cutaneous melanoma, strong ALCAM staining in primary melanoma tissues is linked to short overall and disease-free survivals [87]. Interestingly, the study found that in metastatic lymph nodes, low levels of ALCAM staining are also linked to short overall survival.

### 6.18. Oral Squamous Cell Carcinoma

In a study containing 105 patients with oral squamous cell carcinoma, it was demonstrated that the group presenting with ALCAM cytoplasmic staining and negative E-cadherin membrane staining had a markedly reduced overall survival of the patients [50]. Likewise, the group of patients with cytoplasmic/nucleus beta-catenin and cytoplasmic ALCAM staining also linked to nodal metastasis and late stage of the cancers [50]. Other work has demonstrated that 47% of OSCC tumours stained positive for ALCAM [16] and that the positive staining was linked to a short survival of the patients, appearing to be an independent factor. This is in line with findings from SCC of the head, neck, and pharyngeal cancers in which high ALCAM presents a poor prognostic indicator for the patients.

### 6.19. Bladder Cancer

In a comprehensive analysis of ALCAM in bladder cancer tissues, serum, and urine, it was found that ALCAM staining exhibited a progressive decrease with increased tumour stages [73]. While both serum and urine soluble ALCAM had a marked increase in patients with bladder cancer, the levels of urinary soluble ALCAM revealed a significant correlation with overall survival and tumour stage.

## 7. ALCAM and Cancer Metastasis

Acquisition of invasive phenotypes and metastatic dissemination of cancer cells are key hallmarks of cancer, and cancers with these hallmarks represent poor clinical outcomes of the patients. There is a significant clinical need to fully understand the process of metastasis, identify novel and specific biomarkers and develop novel therapies to treat metastasis or prevent metastasis in those patients with high risk. ALCAM is one of the few cell adhesion molecules detected in tumour-endothelial cells [117]. Additionally, work by Kulasingam et al. has measured the serum levels of ALCAM in 150 patients with breast cancer vs. control groups (100 healthy women and 50 men), along with two other tumour markers, CEA (carcinoembryonic antigen) and CA15-3 (cancer antigen 15.3) [118]. Circulating ALCAM appears to be a better diagnostic marker than the others for the patients. Other work by Ihnen et al. [119] involved 29 patients who died of breast cancer and their tissues collected from autopsies (*n* = 84 in total), including the primary tumours and the matched multiple metastatic tumours. This study [119] has shown that the levels of ALCAM in primary tumours and metastatic tumours correlated exceptionally well (r^2^ = 0.504, *p* < 0.001) and that metastatic tumours in different sites (liver, lung, bone, brain, and lymph nodes) stained reasonably similar to primary tumours. Given the implications of ALCAM in cancer biology and its role as an adhesion molecule, investigations have focused on its clinical usefulness and importance in metastatic progression. The following section outlines the implications of ALCAM in a number of metastases.

### 7.1. Liver Metastases

Bartolomé and colleagues [37] have recently reported that ALCAM, via its interaction with SOSTDC1 (sclerostin domain containing-1), increases the hepatic metastases from colorectal cancer. This interaction was thought to be via an ALCAM binding site, similar to that seen in CD6 of the SOSTDC1 protein. In this complex cellular event, the SOSTDC1-ALCAM interaction is likely to involve PI3K/AKT, α1ß1 integrins, and the src kinases [37].

### 7.2. Brain Metastasis

Injections of tumour cells to induce brain metastases triggered an early rise of ALCAM in endothelial cells, together with other cell adhesion molecules VCAM1 (vascular cell adhesion protein 1), ICAM1 (intracellular adhesion molecule 1), VLA4, and integrin-beta4 [120]. This rise likely assists the endothelial-tumour interactions for seeding and settling of cancer cells in the brain and is supported by additional evidence that anti-ALCAM antibodies significantly inhibited the number of metastatic tumours in the brain [120]. Other works by Münsterberg and colleagues [11] have shown that, compared with primary NSCLC, metastatic brain tumours and metastatic lymph nodes had a marked increase in ALCAM staining. High levels of ALCAM in primary tumours and in brain metastatic tumour are both associated with poor survival of the patients. Lung cancer cells, after knocking down ALCAM, showed more regular presence in the central ventricle and produced high frequencies of metastatic brain tumours compared to control cells [11]. The role of ALCAM in brain metastasis is intriguing, and it has been suggested that raised ALCAM in foreign cells (to brain tissues), for example, lymphocytes, is key for the cells to pass through the BBB (blood-brain barrier), whereas brain endothelium ALCAM appears to play a less important role in this process [121]. This is partly reflected by an in vivo investigation where reduction in ALCAM by selenoglycoprotein would render a greatly reduced presence of metastatic tumour cells in the brain and reduction in brain metastasis [122].

### 7.3. Lung Metastases

Ishiguro and colleagues have stained 147 non-small cell lung cancers and reported that membrane staining is seen in almost half of the tumours and that it is the membrane ALCAM that constitutes a poor prognostic indicator for the overall survival [65]. However, the same authors did not find any significant implication for the cytoplasmic staining of ALCAM (seen in 38.8% tumours) in clinical and pathological factors [65]. Deficiency of stromal ALCAM has been indicated in aiding the homing of cancer cells to lung and lung tissues and accelerating both spontaneous and systemic metastasis [123]. King et al. [26] have shown that breast cancer cells, when overexpressing ALCAM, are increasingly trapped in the lung vasculature, partly reflected by an increase in tumour-endothelial interactions, which can be reduced by anti-ALCAM antibody [26,124].

### 7.4. Skin Metastasis

Ihnen and colleagues have investigated the possible role of ALCAM in different sites of metastasis from breast cancer, namely skin, liver, bone, brain, and lung, and have discovered that it is the skin metastatic legions that have the highest ALCAM staining, arguing a role for ALCAM in site-specific metastasis [125].

It has been shown that the ALCAM present on activated endothelial cells interacts with L1CAM in breast cancer cells, mediating tumour-endothelial interactions [31].

MicroRNA-192 and microRNA-215 are suppressors of ALCAM expression in gastric cancer cells [19]. ALCAM, together with integrins (ITGA5), acts as a key cell surface molecule in cancer cells (melanoma and breast cancer cells, for example) and mediates the intravasation and extravasation during the metastatic process [126]. In this case, the pro-metastatic miR-214 and anti-metastatic miR-145b act as a dual in down- or upregulating ALCAM in cancer cells in this process [126]. In melanoma, miR-214 has also been shown to upregulate ALCAM expression [127]. Likewise, expression of an ALCAM transcription suppressor, together with a reduced ALCAM level in Ewing sarcoma, results in patients with marked decreases in metastasis-free survival [93].

## 8. Circulating and Soluble ALCAM

ALCAM cleavage has been reported to occur through enzymes including MMP14 (matrix metalloprotease 14) and ADAM17 (a disintegrin and metalloprotease 17) [72,128]. Soluble ALCAM is able to antagonise the naturally occurring and natural ALCAM in melanoma cells, a contrast effect to the role of an NH2-terminally truncated, transmembrane variant (ΔN-ALCAM) [129,130]. It also suppresses the action of MMP2 in the tumour cells [126,127]. Given the significant implications of ALCAM in cancer, the detection of soluble ALCAM as a potential serum biomarker (or through other minimally invasive detection of ALCAM) has gained much scientific interest and focused investigation. Raised serum ALCAM levels in patients with oesophageal squamous cell carcinoma indicated a poor overall survival, although this is not seen with recurrence-free survival [60]. In addition, the changes do not appear to be linked to the survival in patients with oesophageal adenocarcinoma [44]. In patients with ovarian cancer (*n* = 61), serum levels of ALCAM are higher than controls and significantly correlated with protein marker CA125/MUC16. This high level is seen in aggressive tumours and high stages. ALCAM levels in ascites are found to be significantly higher than in serum [131]. Additionally, in thyroid cancer, increased serum levels of ALCAM have been seen in patients with aggressive tumours and with lymph node metastases [132]. In patients with prostate cancer, those with metastasis, with nodal positive tumours, and, in particular, those who died of prostate cancer had significantly higher levels of circulating ALCAM. This links to the other important clinical factors, namely PSA and Gleason score of the patients; patients with high PSA levels and high Gleason scores also had high levels of serum ALCAM [79]. Perhaps the most important finding of the study was that circulating ALCAM, similar to PSA, is a prognostic indicator for the patients [79]. In patients with pancreatic cancer (*n* = 115), the levels of circulating (soluble) ALCAM were found to be significantly higher than those with chronic pancreatitis and than the control individuals [62]. Interestingly, there appears to be a diagnostic value of pancreatic cancer (RUC = 0.695). Soluble ALCAM has also been detected in the uterine aspirates of patients with endometrial cancers [69]. The levels of uterine soluble ALCAM appear to be a suitable indicator of tissue invasion of endometrials [69].

Patients with multiple metastatic diseases have been shown to have a markedly elevated serum ALCAM compared with those patients who had single brain metastasis and compared with patients with non-metastatic NSCLC [11]. However, the elevation of serum level does not appear to have any relationship with clinical, pathological, and survival factors, possibly suggesting that the elevated ALCAM in the circulation may, in part, be a reflection of the tumour burden, in addition, to shedding, an interesting lead to follow in future studies. ALCAM-iso2, the variant of ALCAM missing the membrane-proximal region due to exon 13 splicing, is 10 times more likely to shed in response to metalloproteinases (i.e., MMP-14), resulting in more aggressive and metastatic phenotype in vitro and in vivo [72]. The variant form confers cells with lower cell-cell adhesiveness and renders more disseminating and metastatic phenotype [72]. Patients with bladder cancer have been found to have markedly increased levels of soluble ALCAM in the circulation. However, it has been shown that urinary soluble ALCAM showed the greatest increase, namely a 17 times increase over the control group [73], and urinary ALCAM levels have demonstrated a correlation with the overall survival of the patients.

In patients with gastric cancer, serum levels of ALCAM were significantly raised compared with the control group. Although the circulating levels did not show any prognostic links with the overall survival of the patients, raised levels, however, appear to have a value in assessing patients’ response to chemotherapies (*p* < 0.003) [133]. A particular group of patients, namely Saudi patients with breast cancer, were found to have markedly increased levels of soluble ALCAM compared with control. The rise appears to have significant diagnostic value, particularly for high grades of breast cancers, a similar weighting to that of CEA in the same patient group [59]. Another breast cancer-related study demonstrated that in patients with breast cancer (*n* = 157), the serum levels of soluble ALCAM were significantly higher than control individuals, and the levels correlated with shorter disease-free survival, although not with other clinical and pathological factors [134]. The study has reported that there is no correlation between serum ALCAM and tissue ALCAM protein/mRNA levels, indicating non-transcription and non-translational contribution to the raised serum levels, which was possibly due to protease cleavage of the protein, as later demonstrated in many other studies. In patients with hepatocellular carcinoma, circulating ALCAM was markedly raised compared with controls and, interestingly, much higher than those with patients who carried other malignancies, e.g., breast and other GI cancers. It was suggested to be a surrogate marker for complete removal of liver tumours and a superior diagnostic marker over the classical marker AFP (alpha fetoprotein) [89]. There is a lot to learn here before a firm claim is established. Notable points include: ALCAM is more ubiquitously expressed and at suitable levels across the body in a variety of cells and tissues; ALCAM is seen to rise in most cancers; ALCAM circulation is also seen in other non-cancerous conditions; ALCAM has a high baseline level in the normal population and in HCC. Together, they make the usefulness of ALCAM as a diagnostic marker more challenging. However, it is quite plausible to use the molecule as a recurrence or assessment tool after surgery. This seems more possible with the recent development of the device, SiNW-on-a-chip biosensor, to detect ALCAM in serum in as short as 30 min [135].

## 9. ALCAM and Diagnostic and Therapeutic Considerations

The previous sections have outlined the significant implications of ALCAM in clinical cancer and its relation to patient outcomes and prognosis. While the current review focuses strongly on the impact of ALCAM in clinical cancer. There are many important studies and work that focus on its cellular impact together with establishing potential mechanistic aspects. Collectively, this has demonstrated the significance of ALCAM in this key area and led to its potential diagnostic and therapeutic applications.

Wiiger and colleagues [136] have identified a single-chain antibody, scFv173, which recognised ALCAM and was able to reduce ALCAM-mediated cell adhesion and the growth of breast tumours in vivo. Other work using ALCAM as an imaging target tool has been recently attempted. Zarghami and colleagues have conjugated iron oxide microparticles to anti-ALCAM (mouse) antibody and used the conjugate as an imaging tool to detect brain metastasis from various tumour types [137]. The conjugate injection has resulted in the detection of small metastatic foci from the breast, melanoma, and lung cancers on MRI, as hypodensity regions. The overall specificity and sensitivity were at 86% and 79%, respectively, an observation confirmed by immunohistochemical analyses. The study further demonstrated that the approach is more sensitive for metastatic brain tumour from the breast cancer model, compared with that from lung and melanoma, suggesting that there is indeed tumour selectivity. It appears that the main source of ALCAM in brain imaging and histology analyses is likely to be endothelial cells, and normal brain tissue appears to be negative, emphasising the relatively exclusive nature of ALCAM in the cell type. The other interesting finding is that the blood-brain barrier appears to be intact as the metastatic legion had virtually no trace from the conjugate [137]. This is a very interesting finding, however, with some limitations. The primary limitation is the species of the model, namely human cancer cells/tumours vs. mouse tissues. It is well established that ALCAM distributions in tissue and organs are diverse and with a suitable degree of ubiquity. It is therefore anticipated that in a model when both tumours and surrounding tissues are from the same species, namely murine tumours in a murine model, or indeed human metastatic tumours, the antibody would recognise both the endothelial cells and tumour cells. This may have a significant impact on sensitivity and specificity. Nonetheless, the study by Zarghami et al. has provided some very useful information to explore the use of ALCAM as an imaging target tool and possibly for targeted therapies. Additionally, ALCAM has been indeed used as a target in immunoimaging. Tavare’ et al. have constructed conjugates to anti-ALCAM immunoglobulin for the purpose of PET (positron emission tomography) imaging and found that different linkers in length for ^64^Cu-DOTA (1,4,7,10-tetraazacyclo- dodecane-1,4,7,10-tetraacetic acid) delivers diverse tissue tumour profiles of taking up and half-life in vivo [138,139].

Targeting ALCAM as a means of therapy presents new opportunities. However, it needs careful crafting, owing to the diverse role and difficulties in targeting cancer. A suitable reason for this is the contrasting role of ALCAM in cancer; for example, it may affect the growth and colony formation on the one hand but affecting migration and metastasis in an opposite way [10].

### 9.1. ALCAM as a Therapeutic Target

A recent report by Lee and colleagues [140] has shown that using ADC (antibody-drug conjugate) approaches by producing a bispecific designed antibody conjugate of anti-EphA2 and anti-ALCAM was able to trigger a rapid internalisation of the conjugate at the particular ratio. An interesting observation is that when the ADC conjugated with a toxic drum (MMAF, Monomethyl auristatin F), the ADC-drug produced some impressive toxicity in cells that expressed a high level of ALCAM [140]. This is an interesting approach, although the overall impact of targeting/internalising ALCAM itself on the fate of a cell and potentially in the body yet have to be fully assessed. Using an in vivo model, Wrobel et al. has infused mice with selenium-enriched glycoproteins (selenoglycoproteins). Brain metastases in selenoglycoprotein fed mice had markedly reduced brain metastasis and tumour intra- and extravasation in brain vasculatures, which appears to be a result of reduction in ALCAM and PECAM1 in the mice [122]. Other work has shown that an antibody to ALCAM can recognise well endothelial cells surrounding metastatic tumours of the brain [137], indicating a cell-based targeting possibility, namely targeting tumour-associated endothelium. However, a great deal has yet to be learned here. Another recent report by Darvishi et al. [141] has shown that a recombinant protein that has anti-ALCAM scFv feature was able to suppress the growth of tamoxifen-resistant MCF-7 cells and synergistically enhance the anti-tumour effect of tamoxifen. The same was observed with MDA MB 231 cells. Additionally, ALCAM has been shown experimentally to be a possible target in CAR-T-based immunotherapies of osteosarcoma [142]. In ovarian cancer, suppression of the glycosylation of ALCAM by kifunensine was shown to result in the reduction in membranous ALCAM and increase in the cytoplasmic fraction of ALCAM, which coincides with the decreased sensitivity to chemotherapy drugs, cisplatin for example, the work of [13]. Additional work has also demonstrated, in in vivo tumour models, that cyroablation of melanoma resulted in the expansion of ALCAM-positive cell populations, which were comprised of CD45 ^−^ mesenchymal stem/stromal cells, CD11b ^+^Gr1 ^+^ myeloid-derived suppressor cells, and CD4 ^+^Foxp3 ^+^ regulatory T cells [143]. These cells limit the efficacy of cryoblation therapy, further arguing the possibility of targeting ALCAM as a useful way to overcome the resistance to cryotherapy or increase the sensitivity of the treatment.

### 9.2. Truncated ALCAM

Truncated ALCAM, also referred to as soluble ALCAM, has been indicated in possible therapeutic applications in cancers in which ALCAM is a promoting factor in their development and progression. Kinoshita et al. [144] have reported that when soluble ALCAM, in the name of extracellular soil signal sensor receptor, was conjugated with a human IgG2-Fc in order to extend the half-life of the protein, it is able to suppress the lung tropic metastasis of melanoma cells [144].

### 9.3. Targeting ALCAM Partner Proteins

Targeting proteins/enzymes that cleave ALCAM has been a topic of research. Studies by Nuti et al. have identified a new small compound (compound-21) from arylsulfonamide inhibitors that substantially inhibited ADAM17, which in turn reduced the shedding of ALCAM from cancer cells [145,146]. A small non-coding RNA, miR148b, has been reported to be able to directly suppress the expression of ALCAM [147]. When miR145 was conjugated to the AXL tyrosine kinase receptor, it led to the binding of the conjugate to the receptor [148]. This has lead to suppression of the expression of ALCAM and tumour growth in 2D and 3D cell models. Furthermore, Jin et al. [149] have demonstrated that omeprazole, an H+/K+ ATP pump inhibitor of parietal cells of the gut, is able to decrease the expression of ALCAM in pancreatic cancer cells, an effect that appears to be attributable to its interaction with the aryl hydrocarbon receptor, AhR.

### 9.4. ALCAM as Therapeutic Response Indicator

Ma et al. [89] have shown that patients with hepatocellular carcinoma had markedly higher levels of circulating ALCAM. Following surgery, a dramatic reduction in the soluble form of ALCAM in the serum was observed, overall arguing a possible role of serum ALCAM as a possible surrogate marker in response to surgery or the degree of removal of tumours by surgery. McRobb and colleagues [150] have recently shown that ALCAM and CRYAB (Crystallin-Alpha-B) are two endothelial proteins that respond to radiation. ALCAM responded to radiation by translocating from the lateral intercellular space to the apical surface of the endothelial cells, further arguing a possible and prospective target for targeted therapies. Furthermore, Sim et al. has shown that, in rectal cancer patients, ALCAM in pretreatment tumours is an important prognostic factor, however not an indicator to the response to the neoadjuvant therapies (5-FU) [39]. The study has found that ALCAM does indeed respond to chemotherapy.

Roth et al. [151], by developing an anti-ALCAM scFv-liposome, has managed to enable a target drug delivery (including topotecan, doxorubincin, etc.) to prostate cancers with increased efficacy and specificity. Similarly, an anti-ALCAM antibody is conjugated with polymerised liposomal nanoparticles, and the immunoconjugate is used for packaging chemotherapeutic agents, namely doxorucin. This was used to deliver the drug to osteosarcoma cells and in vivo osteosarcoma models. It delivered more anti-tumour effects than a non-conjugated drug, arguing a potential prospect of targeted therapy [94]. In patients with breast cancer, those with high levels of ALCAM mRNA and who received chemotherapy had a better survival rate; in contrast, those with high levels but did not receive chemotherapy had a poor survival [54].

### 9.5. ALCAM and Chemoresistance

In pancreatic cancer cells, reduction in ALCAM by RNAi has been shown to confer resistance to chemoagents [97]. In patients with nasopharyngeal carcinoma, the serum levels of soluble ALCAM were significantly altered in those who showed resistance to radiotherapy [80]. In a rare tumour type, namely giant cell bone tumours, ALCAM-positive tumour cells were more resistant to chemotherapeutic agents, including cisplatin, and also more resistant to radiotherapy [71].

Suppression of ALCAM expression by microRNA, miR-9 miR-148a and miR-152, has been shown to sensitise tamoxifen-resistant cells to tamoxifen [152,153]. It has also been shown that ALCAM, together with a few other cell cycle and drug markers, are key markers in resistance colorectal cancer cells (HCT116 and HT29) to chemotherapy drugs [154] and can also be altered in response to capsaicin in bladder cancer cells [155]. Su and colleagues [156] have reported recently that ALCAM is correlated with drug, i.e., cisplatin, resistance. The study has identified that a majority of non-small cell lung cancers (24 out of 25) had a population of cells that were ALCAM-positive but SLC27A2-negative. The SLC27A2-negative and ALCAM-positive cell populations seem to correlate well with the chemosensitivity and poor survival of the patients, again indicating the nature of ALCAM as a candidate surrogate marker for patients’ response to treatment. Additionally, ALCAM is one of the few mitochondrial proteins highly raised in an ovarian cancer cell line that is resistant to cisplatin (A2780-CP20) [157]. Furthermore, it has been demonstrated that, in patients with cervical cancers, that overexpressed ALCAM protein responded well to chemotherapy and chemoradiation therapies and had a better prognosis [96].

Thus, there are important findings supporting the therapeutic value of using ALCAM either as a target in tumour types where ALCAM contributes to the development and metastasis. On the other hand, soluble ALCAM (truncated ALCAM) is also an option to explore in these cancers. The finding that ALCAM is a suitable indicator for patients’ response to drug treatment is very interesting, although the finding has so far been limited to a small number of cancer types. Wider investigations on other cancer types would be necessary.

## 10. Conclusions and Perspectives

Given its important role and involvement in cancer progression and development, ALCAM has drawn significant scientific interest and research over the past several decades. Its implications in cancer progression and its potential as a diagnostic/therapeutic biomarker have been fully supported by both clinical observations and laboratory studies.

However, there remain some significant challenges in the role of ALCAM in clinical solid cancers. 1. The diverse roles of ALCAM in different cancers, namely tumour suppressive in some cancers, while oncogenic in others. There appears to be an indication that in endocrine-related cancers, including breast, prostate, thyroid, neuroendocrine tumours of the pancreas, and ovarian cancer, ALCAM acts as a favourable indicator (as a tumour suppressor) for the patients. The endocrine link of ALCAM in tumour and metastasis received further support from our recent study on another endocrine tumour, pituitary tumours, which reports that pituitary tumours with low levels of ALCAM are prone to invasion and destruction surrounding bone tissues (Yang Y et al., presented to EACR annual congress 2021). On the other hand, in squamous cell cancers, e.g., melanoma and gastrointestinal cancers, ALCAM serves as an opposite indicator (an oncogene), as high levels of ALCAM are linked to poor clinical outcomes. The reasons for the tumour type-related sharp contrasting outcomes are not clear, but it is likely to be linked to factors ranging from hormone regulation, the cellular location of ALCAM in the tissues, and co-expression of ALCAM with other tumour markers, including hormone receptors and oncogenes such as RAS. 2. Soluble ALCAM (sALCAM) vs. mature ALCAM. ALCAM is a factor contributing to the dissemination of cancer cells and the development of cancer metastasis, as evident from clinical and experimental studies. The clinical studies have indicated the pivotal role of soluble ALCAM in circulation. While the findings are interesting, there remain more questions to be answered in this regard, including the impact of circulating levels of ALCAM in a wide spectrum of cancer types and the ratio between circulating ALCAM and tissue (in particular membranous) ALCAM. 3. Cellular location of ALCAM and the impact on cancer cells and tumour progression. In cells, ALCAM is known to present as a membranous protein or a cytosolic protein. There has been an indication that the cellular location of the protein may have differing impacts on cancer, namely in thyroid cancer and prostate cancer. This is interesting but will require substantial work to firmly establish if this is a wide and common phenomenon in cancer. 4. Targeting ALCAM as a therapeutic option. Perhaps the other most exciting finding is the therapeutic value of targeting ALCAM and the value of ALCAM as an indicator of response to treatment. This is a fertile area to explore, from searching for small molecule inhibitors, developing antibodies to using ALCAM fragments (including soluble ALCAM) to suppress ALCAM in suitable cancers in which ALCAM leads to a poor clinical outcome. Owing to the nature of its cellular surface expression in most cases, ALCAM has been tested as an imaging tool for the diagnosis of both primary and secondary tumours.

Collectively, while much has been elucidated in this field, questions remain, and similarly, areas of contrast exist within the literature. Further investigations into such issues are needed in order to overcome these potential barriers to the clinical benefit derived from ALCAM.

## Figures and Tables

**Table 1:** ALCAM expression in tumours.

Tumour Type	Study Methods	Findings	References
Colorectal cancer	IHC (*n* = 111)	Both membranous and cytoplasmic staining of ALCAM are seen in colon cancer tissues. However, membranous ALCAM is linked with shortened survival of the patients.	[36]
	IHC (*n* = 9 pairs)	Tumour tissues showed highly stained ALCAM compared with normal tissues.	[37]
	IHC and PCR (*n* = 58)	ALCAM-positive staining is seen in 43%. ALCAM-negative tumours had greater incidence of lymph node metastasis, a link greatly increased when there is concurrent KRAS mutation.	[38]
	IHC (*n* = 112)	High levels of ALCAM were associated with longer recurrence-free survival.	[39]
	Gene expression (*n* = 250), IHC (*n* = 105) and ELISA (*n* = 91)	ALCAM is highly expression in tumour tissues and is linked to shorter survival of the patients.	[40]
	IHC (*n* = 299)	More than 70% and 60% of primary and secondary tumours stained positive for ALCAM, respectively. Positive ALCAM staining is a positive prognostic factor for the patients.	[41]
	Gene array (*n* = 64)	ALCAM transcripts were significantly raised in colorectal tissues compared with normal tissues.	[42]
	Germline polymorphism of peripheral blood of patients who resisted 5-FU therapies (*n* = 234)	Polymorphisms of ALCAM, along with LGR5, CD44, and ALDH1, form an independent signature in predicting the time to recurrence of patients with colon cancer who received 5-FU-based chemotherapies.	[43]
Metastatic liver tumours from colorectal cancer	Paired primary and secondary (*n* = 9 pairs), IHC	Stepwise increase in ALCAM protein from normal colorectal tissues, primary colorectal cancer tissues to metastatic liver tumours.	[37]
Oesophageal SCC	IHC (*n* = 299)	High levels of ALCAM in primary tumours associated with recurrence-free and overall survival of the patients.	[44]
	IHC (*n* = 65)	ALCAM staining was seen in 87.69% of tumours, compared with negative staining in control normal tissues. ALCAM linked to tumour grade, TNM staining, and survival.	[45]
	IHC and PCR (*n* = 65)	ALCAM expression is increased in SCC compared with control tissues, and the increase is seen with nodal metastasis and late clinical stages.	[46]
Laryngeal, head, and neck SCC	IHC and RNAseq (*n* = 44) and gene array	Membranous staining of ALCAM. High levels of staining associated with shorter survival of the patients.	[47]
	IHC (*n* = 400)	A total of 70% of HNSCC tumours stained positive for ALCAM, including 12.4% membranous, 40.1% cytoplasmic, and mixed membranous/cytoplasmic in 17.9%.	[48]
	IHC (*n* = 96)	Patients with high levels of ALCAM staining in HNSCC (*n* = 96) had shorter survival. Primary HNSCC (*n* = 68) had significantly lower ALCAM staining than the recurrent HNSCC (*n* = 36) tumours.	[49]
Oral dysplasia and cancer	IHC (*n* = 105)	ALCAM total and cytoplasmic staining were correlated with loss of membranous E-cadherin and beta-catenin in oral squamous cell carcinoma. ALCAM expression together with cytoplasmic/nucleus beta-catenin is an indicator of nodal metastasis and late-stage tumours.	[50]
Oral melanoma	IHC (*n* = 35)	ALCAM-positive staining in oral melanoma is associated with vascular invasion.	[51]
Non-small cell lung cancer and brain metastasis	IHC (*n* = 143 comprised of 51 primary NSCLC, 15 metastatic nodes and 76 metastatic brain tumours)	Metastatic brain tumours and metastatic lymph nodes stained higher for ALCAM compared with primary NSCLC. High staining in NSCLC and in metastatic brain lesions associated with poor survival.	[11]
Breast cancer	IHC and QPCR (*n* = 120 tumour and 32 normal)	ALCAM transcript expression was lower in tumours with lymph node metastasis compared to those without. ALCAM levels were also lower in high-grade/TNM stage compared to lower stage/grade samples. ALCAM levels were lower in those with poorer outcomes.	[52]
	IHC (*n* = 162)	Cytoplasmic staining of ALCAM is associated with shorten survival, nodal status, and early recurrence.	[53]
	Protein blotting (*n* = 160) and gene microarray	Neither protein nor mRNA expressions are linked to pathological factors. Protein ALCAM is seen more in ER-positive tumours. High levels of ALCAM mRNA, when receiving chemotherapy, have better clinical survival, but those who did not receive chemotherapy had worse survival.	[54]
	Gene microarray(*n* = 481)	Low ALCAM expression, together with the status of ER, Her2, and osteopontin, identified a set of patients with markedly shorter survival from three separate cohorts with combined number of 481 patients.	[55]
	GWS (6,669)	ALCAM is a new foci associated with breast cancer (Japan), and together with CLIC6-RUNX1, it makes susceptibility SNPs of breast cancer.	[56]
	IHC (*n* = 153)	ALCAM is largely membranous when present. Seventy out of 153 stained positive for ALCAM and the remaining 83 negatives, and the staining has an intimate relationship with the levels of Wnt5a.	[15]
	IHC and microarray (*n* = 110)	Patients with high levels of ALCAM transcript had longer disease-free survival. The correlation is more significant in patients with high levels of membranous ALCAM and high levels of mannonidase (MAN1A).	[57]
	Transcript analysis and IHC (*n* = 47)	Breast tumour tissues had higher levels of ALCAM gene methylation than normal tissues, and the raised ALCAM gene methylation was seen with lower levels of ALCAM transcript.	[58]
	IHC (*n* = 161)	Patients had highly raised circulating levels of soluble ALCAM and had a potential diagnostic value of breast cancer amongst the particular ethnic population (Saudi patients).	[59]
	IHC (*n* = 2,197)	Reduced/loss of ALCAM staining was seen in most tumour types and was linked to high tumour grade and poor OS and RFS.	[60]
Pancreatic cancer	Circulating cancer cells (CTC) (*n* = 20)	Patients with circulating cancer cells showing high levels of ALCAM had shorter survival.	[61]
	IHC (*n* = 264) and ELISA (*n* = 116)	At the tissue level, there was no significant correlation between tumour grade and staging. However, the circulating levels of ALCAM were significantly higher than the control and those with pancreatitis.	[62]
	IHC (*n* = 97)	Patients who died of pancreatic cancer had high levels of ALCAM staining in pancreatic cancer cells.	[14]
	IHC (*n* = 98)	A total of 12% of pancreatic cancers were positive for ALCAM staining, compared with none in normal pancreatic tissues.	[63]
Ampulla of Vater	IHC	In a rather large series of this uncommon cancer, there was a progressive increase in ALCAM staining from normal mucosa (*n* = 152) to adenoma (*n* = 111) to carcinoma of ampulla of Vater (*n* = 175).	[64]
Lung cancer	IHC (*n* = 147 NSCLC)	Membrane ALCAM staining is seen in 44.9% of NSCLC tumours and is an independent prognostic factor for shorter overall survival of the patients.	[65]
Small intestinal adenocarcinoma	IHC (*n* = 191)	A total of 42% of the tumours stained positive.	[66]
Ovarian cancer	IHC (*n* = 204)	Concurrent high levels of ALCAM and mannosidase MAN1A1 linked to shorter relapse-free survival of the patients, yet low levels of MAN1A1 and high levels of ALCAM linked to better survival of the patients.	[13]
	IHC (*n* = 109)	Cytoplasmic staining or loss of membrane staining of ALCAM is a prognostic factor for patients with ovarian cancer.	[67]
Endometrial cancer	IHC	For early endometrioid endometrial cancer (*n* = 174), positive ALCAM (76.2%) is seen in patients with short recurrence-free survival. Of all the tumours (*n* = 116), 67.4% stained positive for ALCAM with remaining negative. Weak or negative staining was seen at the invading front of cancer tissues.	[68,69]
Oral squamous cell carcinoma (OSCC)	IHC (*n* = 41)	Staining of ALCAM on the membrane of the leading front of the SCC cells was seen in tumours with node involvement and high tumour grade.	[70]
	IHC (*n* = 101)	Less than half (47.5%) of the oral SCC tumours stained positive for ALCAM.	[16]
	IHC (*n* = 107)	Membranous and cytoplasmic staining was seen in OSCC. Cytoplasmic staining was associated with clinical outcomes and survival of the patients.	[32]
Giant cell bone tumours	IHC (*n* = 64)	Patients with high levels of ALCAM staining in the giant cell tumour had shorter disease-free survival.	[71]
Bladder cancer	TCGA analysis and cell work	Bladder cancer cells and bladder tumour tissues expressed high levels of an ALCAM variant (ALCAM-iso2), which is subject to easy shedding in response to MMP14.	[72]
	IHC (*n* = 198) and ELISA (*n* = 120)	Bladder tumours had reduced ALCAM staining with increased staging. Both circulating and urinary soluble ALCAM seen to markedly increase in patients with bladder cancer.	[73]
Thyroid cancer	IHC (*n* = 158)	Total ALCAM in poorly differential thyroid tumours was markedly lower than those with well/moderately differentiated tumours, and the reduction was associated with distant metastasis and shortened survival.	[74]
Prostate cancer	IHC (*n* = 54 pairs)	Over 80% of tumours have raised ALCAM staining, which is largely seen in low grade and low Gleason scores.	[75]
	IHC (*n* = 2,390)	Approximate 70% had membrane ALCAM staining. High ALCAM expression was associated with less aggressive tumour phenotypes, pre-operative PSA (prostate-specific antigen) levels, and a reduced risk of biochemical recurrence.	[76]
	Gene microarray and IHC (*n* = 42 pairs)	A total of 86% of prostate tumours are positive for ALCAM and are a prognostic marker for prostate cancer.	[77]
	Public microarray analysis	ALCAM mRNA enhanced in metastatic disease. High mRNA expression of ALCAM corresponded with poor outcomes.	[78]
	IHC (*n* = 48) and ELISA (*n* = 229)	Patients with metastatic disease, nodal-positive tumours, and who died from prostate cancer had high ALCAM staining. Circulating ALCAM has a prognostic value on the survival of the patients.	[79]
Nasopharyngeal carcinoma	ELISA (*n* = 60)	Patients with radioresistant response had high levels of circulating ALCAM. High staining of ALCAM is linked to favourable clinical and pathological features and with low risk of biochemical recurrence of the patients.	[80]
Mesothelioma of the pleural cavity	IHC (*n* = 175)	ALCAM-positive mesotheliomas had a significantly shorter survival of the patients.	[81]
Intracranial meningioma	IHC (*n* = 20)	Meningioma tissues had significantly higher levels of ALCAM compared with normal tissues.	[82]
Neuroblastoma	IHC (*n* = 66)	Weak ALCAM staining is linked to a short RFS and OS.	[83]
Glioblastoma	IHC (*n* = 39)	Tumours rich in ALCAM had shorter overall and disease-free survival.	[84]
Medulloblastoma	IHC (*n* = 45)	Majority of the tumours (67%) were negative for ALCAM staining. The positive stained tumours (18) were seen in the WNT group and SHH group. The presence of ALCAM is associated with CTNNB1 and nuclear β-catenin expression.	[85]
Melanoma	IHC	There is a progressively increased staining of ALCAM from nevi (15%, *n* = 71), primary melanoma (53%, *n* = 71) to metastatic melanoma (69%, *n* = 84).	[86]
	IHC (*n* = 104)	High levels of ALCAM staining seen in patients with shorter overall and disease-free survivals. In addition, low ALCAM staining in metastatic lymph nodes is also seen with shorter overall survival of the patients.	[87]
	IHC (*n* = 110)	A total of 65% melanoma were positive for ALCAM compared with 74% nevi.	[88]
Hepatocellular carcinoma (HCC)	IHC and ELISA	HCC tumours stained more strongly for ALCAM than normal liver tissues, and HCC patients had markedly high levels of circulating HCC.	[89]
	IHC (*n* = 129)	In recurrent hepatocellular carcinoma (RHCC), positive ALCAM was associated with time to recurrence and microvascular invasion.	[22]
Salivary gland tumours	IHC (*n* = 45)	Adenoid cystic carcinoma and mucoepidermoid carcinoma ALCAM staining were markedly higher than benign pleomorphic adenomas and normal tissues. High-grade and late-stage malignant tumours had higher staining than early stages.	[90]
Gastric cancer	IHC (*n* = 142)	Both membrane and cytoplasmic staining are present and linked to nodal metastasis and vascular invasion.	[91]
	IHC and PCR (*n* = 66), ELISA (*n* = 72)	Both protein (IHC staining) and transcript expression of ALCAM were highly raised in gastric cancer compared with control tissues. The patients also had significantly higher levels of circulating ALCAM than controls and those with non-cancerous conditions.	[92]
Ewing’s sarcoma	ChIP-seq and gene profiling (*n* = 98)	Most sarcomas stained positively for membranous ALCAM, and high levels of staining seen in patients with suitable MFS survival. Furthermore, high levels of HDGF (an ALCAM transcription suppressor) and low levels of ALCAM collectively present in patients with the worse prognosis.	[93]
Osteosarcoma	IHC (*n* = 10)	Membrane and cytoplasmic staining are seen in both primary and metastatic tumours.	[94]
Malignant mesothelioma	IHC (*n* = 55)	A total of 55% of the 47 malignant mesotheliomas stained positive for ALCAM. Overexpression is linked to shorter survival.	[95]
Cervical cancer	IHC (*n* = 233)	Over half (58.4%) of tumours were strongly positive for ALCAM.	[96]
